# 4023 Catecholamines and Opioid Therapy Requirements for the Management of Acute Post-Procedural Pain: The clinical Trend to Identify Remarkable Elements in Opioid Drug Dependency

**DOI:** 10.1017/cts.2020.326

**Published:** 2020-07-29

**Authors:** Armando Uribe Rivera, Ana Carolina Machado Perez, Hans Malmstrom, Yang-Fan Ren, Linda Rasubala, Adam J Carinci

**Affiliations:** 1 University of Rochester Medical Center; 2 Eastman Institute for Oral Health; 3 Anesthesiology Department, Strong Memorial Hospital

## Abstract

OBJECTIVES/GOALS: To compare the opioid drug requirements amongst those individuals with high levels of catecholamines in blood and acute post-procedural pain, by ICD9/10 codes (experimental) to those with normal levels of catecholamines and acute post-procedural pain (AP-PP) only (controls) METHODS/STUDY POPULATION: In collaboration with both the Informatics and the Biostatistics Departments at CTSI and under the auspices of the IRB at the University of Rochester, we completed the collection of ~8,000 electronic health records(EHRs) of adults 18 years and older with surgical appointments at Strong Memorial Hospital (SMH), who met inclusion criteria, from January 2006 to September 2019 and received Fentanyl therapy for AP-PP management. Subjects were categorized in a two-arm-matched case-control fashion. A ratio of 1(Experimental):1(Control) was utilized. Analytic comparisons were completed using normal distribution statistical methods with p >0.1 for significance. RESULTS/ANTICIPATED RESULTS: After removal of duplicates and exclusion of EHRs, a total of 17 subjects met inclusion criteria for the experimental group. We matched controls (n = 17) with experimental subjects for age, gender and surgical procedure for accurately compare opioid requirements in the postoperative recovery. Mean age of subjects was 69(+/-10.1235) years old. Most of subjects were females (70%). Mean Fentanyl requirement was significantly different in the experimental group 466.17(625.621)mcg compared to 215.58(353.323)mcg in the controls (p value 0.07832) DISCUSSION/SIGNIFICANCE OF IMPACT: It is suggested that healthy individuals with genetic variations in pain pathways including; the COMT and MAOA rendered individuals with higher levels of catecholamines in the body driving abnormal responses to pain sensitivity. We emulated this genetic variation for clinical purposes using ICD10/9 codes of those with conditions related to higher catecholamine levels in the body. Based on our preliminary results, we suggest that COMT and MAOA genetic variations could impact opioid drug use and the current opioid dependency and epidemics in the U.S. This study will address remarkable questions and identify strategies about this topic.

